# Digital Responses of UK Museum Exhibitions to the COVID‐19 Crisis, March – June 2020

**DOI:** 10.1111/cura.12413

**Published:** 2021-04-08

**Authors:** Ellie King, M. Paul Smith, Paul F. Wilson, Mark A. Williams

## Abstract

The impact of the COVID‐19 Crisis on museums and galleries has been paramount, with the sector taking on long‐term recovery plans. This paper examines this crisis in the context of temporary exhibition programmes of UK museums, studying online content for 21 museums with exhibitions due to open between March and June 2020. Analysis was conducted, noting how COVID was considered, how content was presented, and discussing the emerging themes of access, embodiment, and human connection. In considering these results in the context of wider digital heritage literature, several questions are raised in terms of how digital content is conceptualised, presented, and valued. At a crucial turning point in the sector, these aspects will need to be considered as museums and galleries continue to adapt in light of a post‐COVID world where practices, both digital and physical, will undoubtedly shift.

## Introduction

When the COVID‐19 pandemic forced 90% of museums and galleries worldwide to close their doors in March 2020 (UNESCO, 2020), cultural and heritage institutions were left in a difficult predicament. Despite the immediate loss of up to 80% of income (NEMO, 2020) and the fear of more long‐term economic disruption, museums and galleries nevertheless saw themselves as community leaders, bringing people together and, according to John McMahon (2020) of Arts Council England, ‘providing hope – a means of processing uncertainty and trauma and an outlet for grief for families kept apart.’ In practical terms, this meant moving resources and services online, and UNESCO have identified over 800 individual actions by museums and galleries in response to the pandemic (UNESCO, 2020).

During this disruption, UK museums and galleries had diverse responses with regards to their temporary exhibition programmes that were due to open during the period of closure. Identifying 88 temporary exhibitions across the UK, various responses from museums and galleries in the wake of the pandemic are noted, later focusing on the 21 museums which provided online content of their planned exhibitions. Analysing presentation style and features, how COVID was discussed, and themes of access, embodiment, and human connection, the responses highlight discussion around the value of online exhibitions. Several questions are then posed concerning how museum digital behaviour may change in the wake of the pandemic, which museums and galleries need to tackle in the future to ensure their role as cultural institutions bringing communities together is continued.

## Background

Since the 2020 lockdown, several organisations have conducted surveys to assess the impact of COVID‐19 on the heritage and cultural sector. Whilst much of the response has focused on the funding needed to keep the sector afloat, organisations such as UNESCO, the *International Council of Museums* (ICOM) and the *Network of European Museum Organisations* (NEMO) have also documented the online activities of museums and galleries during the pandemic. This move comes in the context of both a rise in the level of digitisation of collections and an increase in online offerings of museums and galleries over a number of decades, which has been extensively documented (e.g. Parry, 2005, 2007, 2010; Wellington & Oliver, 2015; Williams, 1987). Within the very specific context of the pandemic however, UNESCO (2020) have detailed five types of activities seen in the rapid development of online presence for museums. These are:

Use of previously digitised resources.

Digitisation of planned activities during the months of lockdown.

Increased activity on social media.

Special activities created for lockdown.

Professional and scientific activities organised in the context of lockdown.

However, this rapid development did not come *ab initio*, with UNESCO (2020) commenting that many of the responses build on ‘investments made before the pandemic’. ICOM (2020) reported a similar story, with digital communication activities increasing for at least 15% of museums, whilst NEMO (2020) reported that 58% of museums held the same activities, with 37% increasing activities, and 23% starting new ones.

This highlights the fact that whilst online content and resources were promoted during museum closures, as would be expected, it was not all created in direct response to the pandemic. However, by studying the actions of museums and galleries in relation to their temporary exhibition programmes in the wake of COVID‐19, and thus focusing in on the second type of activity identified by UNESCO above, we may be able to see a more direct response and immediate reaction to new events where online content had not previously been planned. Subsequently, studying UK museums’ temporary exhibition programmes that were due to open either just before or during lockdown provides the potential to assess a more direct response of museums to the pandemic, with the likelihood that content had not been planned or prepared prior to lockdown. In analysis, several trends of actions are highlighted, including how COVID is discussed, how content is presented, and how online exhibitions are conceptualised more generally. Several questions and discussions subsequently emerge about the role of museums and galleries and the role of both physical and online exhibitions that the cultural and heritage sector will need to consider in their uncertain and changing future.

## Method

In tracking plans for temporary exhibition programmes, we can identify a direct response from UK museums to the halting of their physical activities. Several criteria were implemented in order to collect a representative sample for analysis. Firstly, a range of exhibitions from across the UK regions was sought, including London and eight regions of England, as well as Scotland, Wales, and Northern Ireland. Secondly, the exhibition had to be a temporary programme that was due to open in March, April, May or June 2020. Such a narrow window was necessary in order to capture the direct response to the closure of museums and how museums then reacted to changes to their exhibition programmes. Thirdly, the exhibition subject could not be about COVID‐19 or collecting during the pandemic, as again this does not show the direct change of previously planned activities affected by lockdown.

With these sampling criteria, several internet searches via Google were conducted to identify museum websites which were then explored, often on ‘What’s On’ pages, for information on their temporary exhibition programme. A convenience sampling method was used, firstly using a compiled list of top UK museums by visitor numbers and then searching for museums in various cities and regions of the UK.^1^ Whilst this means that there is no guarantee that every temporary exhibition was identified and sampled, it is unlikely that many important examples have been omitted considering the rigorous search method implemented.

88 temporary exhibitions from UK museums were identified, with a spread across opening month (Table 1) and UK region (Table 2). The sample was then sorted by response based on the information provided on museum websites. Four different actions were identified:

Statement that the museum is closed, but no additional information given about the temporary exhibition.

Statement that the exhibition is extended.

Statement that the exhibition is postponed.

Statement that the museum is closed, but some form of online content of the exhibition is provided.

Full details of the responses are found in Table 3. Just over half of the samples (53.4%, *n* = 47) responded with the first option of closure, and just under a quarter (23.9%, *n* = 21) provided online content.

Having identified 21 museums that provided online content for planned exhibitions, a deeper analysis of this content was undertaken. ‘Content’ was made up of all the text‐based content provided on the temporary exhibition pages, which were then copied into text‐editing documents in order to undertake analysis. All video and audio content was transcribed into text‐editing documents, and images were saved. In the case of exhibitions displayed via virtual reality, the research team explored the programmes and wrote descriptive commentary of their actions and features of the exhibition.

The full details of the 21 online exhibitions and museum names, including URL where available, are provided in Appendix 1: Data Sources. Where a URL is not available, this means that during compilation of references for this research, the webpage hosting the online exhibition was no longer available, or the content of the online exhibition had been taken down due to the museum reopening. The data sources have been numbered, and any further quotes or references used in analysis have been referenced with this number.

## Results

**Figure 1:**
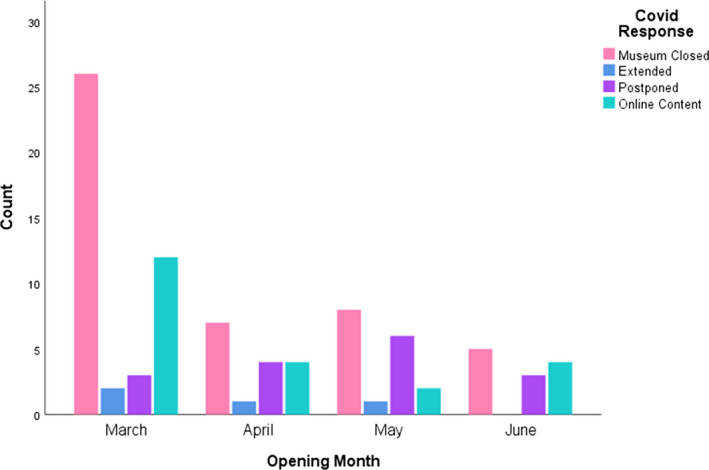
Exhibition Response by Opening Month. Source: authors.

In assessing exhibition responses to COVID‐19 by opening month (Figure 1), there are several trends to note. Firstly, with the majority of exhibition programming due to open in March, it can be seen that there are 26 direct museum closures but also that online content was provided for 12 exhibitions. This suggests that whilst there was an immediate reaction to close and do little else, a number of museums did not want the work that had gone into the exhibition to go to waste and therefore translated content into the online sphere, or were already planning to do so. Secondly, there is a slight spike in postponed exhibitions in May, during a time of lockdown when there was great uncertainty with regard to how long museums would remain closed, and so some museums took the decision to postpone their exhibition programme, and wait until the museum reopened to continue as planned. However, there is also an interesting rise of museums providing online content in the later months of lockdown of May and June, suggesting that time was used to prepare the transfer of exhibitions online. Due to sample sizes, there are no trends to note in assessing exhibition responses by UK region. Further analysis was conducted on the 21 online exhibitions identified, and several trends investigated. Features and types of presentation for the online exhibitions were identified, documenting the various decisions museums had made in developing their online content. A thematic analysis further highlighted several themes of presentation, namely: (a) Talking about COVID, (b) Access, (c) Embodiment and (d) Human Connection, which are discussed below.

### Talking About COVID

**Figure 2:**
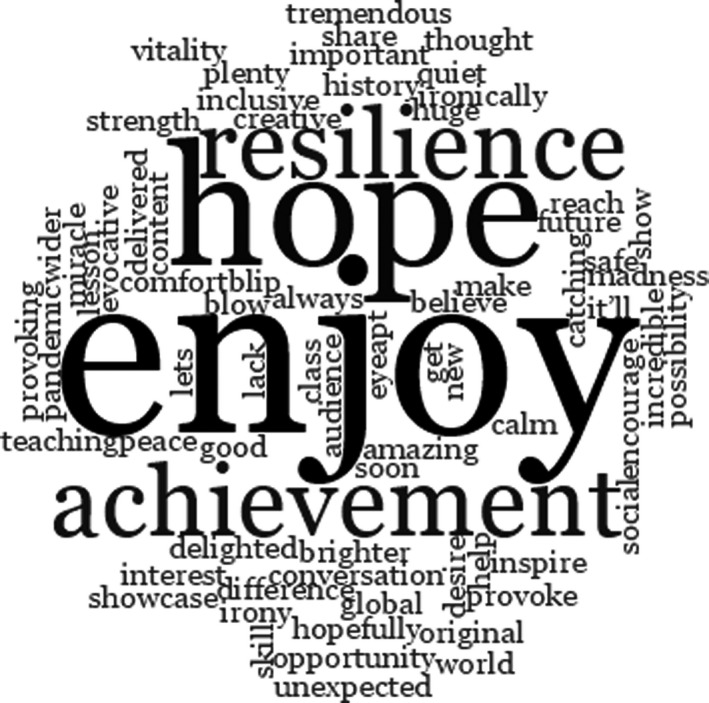
Word Cloud of Positive Terms to Discuss COVID‐19. Source: author.

Whilst specifically referencing the closure of the museum due to COVID‐19, some of the online exhibitions also talk about the pandemic in a way that reinforces their role as museums providing community comfort in a time of uncertainty. Twelve exhibitions explicitly state that the museum is closed, but they often take a mildly optimistic tone, commenting that despite this closure, the exhibition can still be enjoyed online. For example, the Ashmolean Museum commented that ‘our Young Rembrandt exhibition is currently closed, but you can still visit virtually’ [1]. Similarly, of the exhibition due to open at the Sedgwick Museum of Earth Sciences in Cambridge, it is stated that ‘this has been postponed and instead has become our first online exhibition’ [11]. It seems that despite the disappointment of closures, these two museums are trying to make the best of the situation. This optimism can be seen further by looking at the words and emotions used in the online exhibition content to discuss the current situation (Figure 2).

This was performed using QSR NVivo, where words and phrases to describe how the museum and curators felt were interpreted and coded either positively or negatively. A Word Cloud of positive and optimistic comments was then produced to visualise the kind of comments that had been made. Whilst words such as ‘sadly,’ ‘unfortunately,’ and ‘gutting’ are used to discuss the museum closures, there are many more words making light of the situation. Comments like ‘we hope you enjoy the exhibition’ are frequent, the ‘resilience’ and ‘achievement’ shown in the situation is recognised, and the closures are seen as an opportunity to be ‘inclusive’ and ‘creative.’

This reinforces the idea that museums see themselves as playing an important community role. It can be seen through the use of these terms that the museums providing online exhibition content position themselves as a place of escapism, comfort and hope in an uncertain time. In response to the pandemic, museums have emphasised their role as comforting cultural institutions, this time in a virtual space.

In a more direct sense, two online exhibitions discussed how their subject matter provided an opportunity for learning from culture and history in order to better weather the storm of the pandemic. In a curator talk from Madeleine Kennedy at the Guildhall Art Gallery, it is emphasised how the exhibition *The Enchanted Interior* provides an ironic yet appropriate parallel to the lockdown. With the subject of the exhibition being about artwork depicting females entrapped in their homes, Kennedy commented:

*“So given the emphasis that the exhibition had on […] what it’s like to inhabit an enchanted interior […] there is a kind of extra irony that we can now only see the exhibition online or through the exhibition catalogue. But in a way, it’s also quite apt that we can only view this exhibition from the home when so many of its works talk to us about what it’s like when your domestic realm becomes your entire world and you can’t really have much of a life beyond that space […]. And I was thinking about what these works offer us in this really difficult time”* [5].

Similarly, in an introduction to *Lisburn and the Second World War* at the Irish Linen Centre, the Chair of Lisburn Council’s Leisure and Community Development Committee, James Tinsley, commented on the resilience demonstrated during the Second World War, and that ‘history is always teaching us and I believe this is an important lesson that can help us all get through this pandemic’ [21]. The parallels drawn between the war and the pandemic were particularly strong around the 75th Anniversary of Victory in Europe Day on May 8th, where a sense of community spirit and resilience felt in 1945 was reapplied to 2020. This therefore shows us how in talking about COVID, the online exhibitions reinforced their role as an outlet of hope and community in a general way, and some used culture and history to guide communities in current times.

### Access

One evident positive feature of the online exhibitions is the increased access for audiences who would not otherwise be able to physically attend, which was highlighted by several museums. For example, The Hunterian exhibition *A Curator’s Choice* highlighted that ‘this online version allows you to enjoy the best of the show from wherever you are’ [19] and the Aberdeen Artists Society annual exhibition *Coming Home* recognised that ‘it is an opportunity to reach a wider global audience’ [18]. Beyond a larger audience, the lockdown has also provided, according to *Coming Home*, ‘a way of being more inclusive, thus enabling more artists to be selected’ [18] as well as, according to Compton Verney’s exhibition *Cranach: Artist and Innovator* a ‘flowering of curators giving tours of exhibitions’ [2].

Together this suggests that, despite the tragedies of the pandemic, museums have generally treated the lockdown in an optimistic way, highlighting their role as a place of cultural safety, an ability to learn from their stories, and a means to reach a wider audience in a more inclusive and accessible way. In acknowledging this directly in their online exhibition content, the research highlights, first and foremost, an awareness of the role of cultural institutions in society. With more detailed exploration into how this online exhibition content is presented, several themes, issues and questions emerge that seek to realign and redefine this societal role in the future.

### Presentation

The 21 museums that provided online content for their exhibitions did so in a variety of ways. Firstly, there were variations in the use of different features to communicate exhibition subject matter. Nearly all presented content on their own museum websites or on linked microsites, but three exhibitions were presented on social media, namely *Florence Nightingale: Health in the Home* at Pickford’s House, Derby [8] and *Into the Blue* at Tenby Museum, Pembrokeshire [17], who both hosted video content on YouTube, and *The Maguire Story* at Enniskillen Castle, Northern Ireland [20], who hosted content on Facebook. Whilst many other exhibitions did use video, these were embedded into websites and not provided as an external YouTube link.

Of the eighteen museums hosting content on their own websites, all of them used text content and image content, fourteen used video content, and four utilised audio content, such as a podcast. Beyond these features, the overall layout and navigation of the online exhibitions were varied, with content presented in both linear and non‐linear ways. Exhibitions like *Young Rembrandt* at the Ashmolean [1] had a simple one page layout, in which the visitor could scroll down to view content and watch videos. Whilst some navigation between sections of the exhibition was provided (e.g. ‘click to go back to the start of Part 1’) there was little freedom of navigation for the visitor and a structured order of content was presented. In a similar way, some exhibitions had a linear presentation of click through pages, rather than the ability to scroll down on a single page. This was evidenced at Saffron Walden Museum’s exhibition *All Fired Up! A History of Firefighting in Essex* [12]. Again, content was rigidly structured with little freedom for the visitor to navigate out of order.

Some exhibitions took a less linear approach, with a ‘hub and spoke’ model of presentation consisting of a central page with smaller branches of content. Exhibitions such as *Skyscape* at Worcester Museum [9], *Hitchens: Aspects of Landscape* at Southampton [7], *Coming Home* at Aberdeen Art Gallery [18] and *Degrees of Truth* at the Sir John Soanes Museum [3] all presented their content using a ‘hub’ holding page with links to various ‘rooms’, ‘galleries’ or ‘floors’ that the visitor could click on to explore. This was a more free‐flowing approach, with visitors moving back and forth to access content from the main page of the exhibition. Then, at the other the end of the spectrum, were those exhibitions that provided full visitor control through Virtual Reality 360˚ tours of the exhibition space. This was selected for *Blitzed* at Nuneaton Museum [10], and for *Becoming Vanderbilt* at The Breakers, Newport [16]. With full freedom of visitor movement and an ability to click on both objects and text panels to gain more information, the Virtual Reality tours presented content in its closest form to the corresponding physical exhibition that had been planned. It is interesting to note that these two exhibitions opened in May and June respectively, suggesting that it was possible for the content to be produced in this way because of the time allowance the museums had to prepare it.

Overall, the online exhibitions analysed used a variety of different methods and media to present their online content, which gave visitors a range of flexibility and freedom to explore the exhibitions. In noting this, there are three further questions to consider. Firstly, how reactive were these exhibitions in response to the pandemic, or is it feasible that such online content had been planned before museums were forced to close? The digital heritage sphere has grown over the last few decades (Wellington & Oliver, 2015) and so it is likely that at least some of the online exhibition content had been planned prior to COVID‐19, considering not all museum activities during lockdown were new (NEMO, 2020). However, there are some suggestions that if not all of the content was unplanned, at least some of it was. For example, *Dawn of the Wonder Chicken* at the Sedgwick noted that:

*“The museum planned to open a temporary display to coincide with the publication of this work in the March 2020 issue of the journal* Nature. *This has been postponed and instead has become our first online exhibition”*. [11]

This comment suggests that the online content was not previously planned prior to the museum closure. Similarly, Compton Verney’s video tour of *Cranach: Artist and Innovator* stated that ‘This is a virtual tour of the exhibition we created… While it’s nothing like seeing the exhibition in person, until we can, I thought I’d share some of the highlights with you’ [2] suggesting that the physical exhibition was their main intention. However, with the impact of the pandemic becoming apparent, it is likely that such online considerations will need to be made more frequently in future and that creatively agile museums will be successful in making these sharp transitions.

This leads to a second question as to how decisions about presentation are made, but assessments of the final product of the online exhibition in research permit few insights into the decision‐making process of such developments. Discussions of online exhibitions at Gloucester Museum (Taylor‐Jones, 2020), Victoria Gallery and Museum Liverpool (Euston, 2020) and the Royal Armouries Leeds (Ward, 2020) suggest that presentation choices made were *ad hoc*, or driven by available staff skills. As Nigel Taylor‐Jones (2020) wrote for Gloucester,

*“Luckily, there is a great marketing team in the museum who are very comfortable with social media platforms, and they provided me with all the support I needed to develop virtual tours of the museum that could be shared on Facebook and Twitter during our closure.”*

Similarly, Nicola Euston (2020) of Victoria Gallery and Museum advised sector colleagues to ‘find out what skills your team have and provide opportunities to utilise these skills.’

The third question raised concerns the effectiveness of such presentation choices. With little audience testing conducted for the various presentation styles, it is difficult to make any judgements on what works best in this context. The ‘hub and spoke’ model and the virtual reality model may provide embodiment and an essence of the museum online, differentiating the exhibitions from other websites. In an online age where information is readily accessible, it is important to question what makes online museum offerings unique, when information is available from websites such as Wikipedia. If the uniqueness of physical exhibitions is to provide *learning experiences* rather than just information, an online offering which captures a sense of experience, such as in the virtual reality tour, is perhaps best to uphold such a role.

However, whilst these presentation methods will need to undergo further research in the future to assess their effectiveness, this current analysis has identified some common themes that consider the deeper conceptualisation of online exhibitions.

### Embodiment

Perhaps the largest theme to emerge from the study is that of the embodiment of the online exhibitions. Museums have made a significant attempt to make the online exhibition experience more than just browsing a website. Firstly, fourteen of the twenty‐one online exhibition use either the terms ‘visit,’ ‘explore’ or ‘enter’ to describe viewing the exhibition content. This is important: instead of simply being a ‘viewer’, visitors are given a sense of movement and an active journey through the use of these terms, as if they are going somewhere when they enter the exhibition pages. There is a sense of physicality to it, in that visitors are not simply sat at computers reading about the exhibition but have been transported to the museum, albeit virtually, when they ‘visit’, ‘explore’ or ‘enter’.

This is further emphasised by the organisation of content into ‘rooms’ or galleries’, which is done by eight of the exhibitions, especially those using the ‘hub and spoke’ model of presentation. Similarly, sixteen exhibitions made reference to their physical museum counterpart in some way, either through virtual reality tours, video tours of the gallery, or simply photos of artwork in the gallery. This is important, because it clearly embeds the digital exhibition within the physical exhibition and seeks to provide a digital experience that is comparable to the physical experience that visitors would have got if not for the lockdown. There is a clear effort to make the digital exhibition experience more than just scrolling down a page, which in turn raises considerations on what an online exhibition is, and should be.

The concept of embodiment has been explored extensively in previous research on digital heritage. Several researchers have theoretically explored embodiment as the conscious experience of bodily aspects (Johnson 1987; Lakoff & Johnson 1980, 1999) and an engagement that makes our world meaningful (Dourish 2001). Considering this therefore, Rahaman (2018) argued that digital heritage embodiment includes active participation, task accomplishment, and practical action and feedback from end‐users. Similarly, Kenderdine (2015) discussed the embodiment of digital heritage through five ‘bodies’ (Johnson, 2007): the biological, phenomenological, ecological, cultural, and social, which creates an immersive, interactive experience where bodily experience joins together the past and the present. Assessing the COVID content against this previous research, it is evident that the online exhibitions largely do not conform to these recommendations, with only a few examples of interactivity available. Largely, the exhibitions consist of clicking, scrolling, and watching videos. However, with the prevalent use of the embodied language as discussed above, it is clear that museums are trying to create a sense of presence and place (Hall 1969, Slater 1999; Tuan 2001) and are trying to transport online visitors from their homes back into museums. Phenomenological embodiment (Johnson 2007, Kenderdine, 2015) thus plays an important role.

### Human Connection

It is also evident in online exhibition content that there is a heightened prominence of curators, artists, and researchers. In the Ashmolean’s *Young Rembrandt* exhibition [1], curator An Van Camp gives an introductory video tour of the exhibition, and similarly the curator of Compton Verney’s *Cranach: Artist and Innovator* [2] holds a podcast interview discussing the exhibition with the contemporary artists it features. In the *Health in the Home* exhibition at Pickford’s House [8], curator Lucy Bamford discusses some of the exhibition objects in a YouTube video, and for *Dawn of the Wonder Chicken* at the Sedgwick [11], great attention is placed on the research team who made such discoveries, with photographs of the researchers included in the exhibition. In the context of lockdown, where human connection is limited, such emphases are important.

Similarly, there are also direct calls for audience engagement in the exhibition specifically and with the museum at large. For *Health in the Home* the museum commented *‘as part of a public call out, we’re asking What Do You Do to Be Well?’* [8], demonstrating again the community and engagement role that museums play. Similarly, several exhibitions have directed visitors to their social media content, with the Irish Linen Centre asking their audience to *‘please share the exhibition with friends and family, and make sure you tour our #VirtualMuseum’* [21], whilst Compton Verney commented *‘please remember, although we’re not open physically at the moment, we are open virtually, so do visit our website and our social media channels, we’d love to hear from you’* [2]. This social media practice is widespread, with ICOM (2020) stating that over half of museums they surveyed had increased their social media activities.

## Discussion

The themes of access, embodiment, and human connection identified in the exhibition content, lead to more fundamental questions about what online exhibitions are, and should be, conceptualised as. It leads to a question of the value of exhibitions, both online and physical, and we must ask whether there is a trade‐off happening here. Despite the efforts to embed the digital within the physical, making clear references to the gallery space, it is fairly evident that an online exhibition does not provide the same social and embodied experience as the physical museum. There is no travel to get there, no welcome from a member of staff, no opportunities to discuss the exhibition with another visitor, and no opportunity to grab a coffee or browse the gift shop afterwards. There is an evident lack of this social embodiment of a museum visit in an online space. To make up for such losses therefore, we can see an emphasis placed on access and human connection. It is possible to visit exhibitions in places you could not normally visit, especially those in different countries, from the comfort of your own home. Furthermore, content is often presented with links to further information from archives and digitised collections, such as at Compton Verney. There are also comments on behind‐the‐scenes insights, with previously unseen collections being used in *Blitzed* at Nuneaton [10] and *Health in the Home* at Pickford’s House [8], and curator Madeleine Kennedy giving a talk about how *The Enchanted Interior* exhibition evolved over several years at The Guildhall [5]. This suggests that in the absence of a physical museum, online exhibitions are attempting to provide visitors with something exclusive that they would not normally receive from a visit.

This leads to the question as to what is truly meant by our digital practices and what online exhibitions are. The content presented during the pandemic demonstrates that online exhibitions are very much tied to their physical counterparts. This may be a good thing, in attempting to provide that sense of embodiment and physical experiences that museum buildings provide, but with a distinct lack of a comparable experience, is it right that we tie digital practices to physical ones? Writing for Frame, a media brand for interior‐design professionals, Peter Maxwell (2020) commented that ‘clicking through to a page of thumbnails that simply link to hi‐res artwork images doesn’t feel like an event’, thus demonstrating some of the problems of the digital exhibition experience. With the longevity of the pandemic becoming more apparent at the time of writing, digital offerings of exhibitions are likely to become more important for visitor engagement, and thus may need more attention and thought in their development. Anna Ward (2020) of the Royal Armouries commented that in turning a physical exhibition into a digital one, ‘we found that we were able to use the original theming and interpretation hierarchy devised for the physical exhibition to propose our shape for our online offer,’ highlighting how the online exhibition is guided from the physical exhibition. The use of the physical gallery in many photographs and videos of online content, as discussed above, also suggests that the exhibitions studied were largely developed as physical entities first. In contrast, Nicole Meehan (2020) argued for such a rethink of the calibration of digital museum objects, arguing that instead of tying them to their physical counterparts, we should empower them with full value and agency to democratise collecting and interpretation practices. The same could be said for exhibitions, where online manifestations are not simply a second form of a physical exhibition, but instead an exhibition in their own right offering something unique yet equally valuable to museum visitors.

The debate about the relationship between physical and virtual museums has long been discussed within the realm of digital heritage. Beginning with Malraux’s notion of a ‘museum without walls’ (1947), research and debates have centred on how physical museums should relate to digital ones. ‘Can a virtual museum merely be a replica of the physical one, or should it be something radically different?’ asked Huhtamo (2010), with Battro (2010) arguing that the virtual museum had developed beyond its physical counterpart into a different type of museum visit. Alternatively, some research focuses on the complementary relationship of virtual and physical museums, such as Bandelli (2010) who considered the overlap of virtual and physical content, and Galani and Chalmers (2010) who explored social museum visits between both local and remote visitors. What is apparent from this research is that whilst virtual and physical spaces do have uniqueness from each other, they are nevertheless complementary and mutually supportive.

In considering the COVID online exhibitions, there exists only the virtual museum. Visitors can only visit remotely, they do not have a choice to visit in person. In removing access to the physical museum, it is interesting therefore that the digital exhibitions nevertheless still tie themselves to their physical counterparts. Despite having some unique features as discussed above, it is clear that museums still wish to hold on to the physical–virtual overlap. This decision may have been guided by time and resource constraints, and that using gallery photos was the best that could be done given the circumstances, but it is still interesting that, given this rare opportunity, there was not a detailed exploration of the virtual museum in its own right. This may be a direction of future research in the post‐pandemic world.

It is evident that more work needs to be done to truly understand how digital exhibitions work for audiences. Audience research and evaluation has long been common for physical exhibitions, but it is far from widespread for online ones. There are a number of examples of research, such as Kenderdine’s *I Sho U* tool for evaluating the embodied experience (2017); Lin and Gregor’s development of guidelines for learning websites (2006); Rahaman’s framework for digital heritage interpretation (2018); and studies for assessing museum websites (Marty, 2008; Walsh et al., 2020). However, none of these focus on online exhibitions as this work has done. As discussed above, different museums have presented online exhibitions in different ways, but it remains to be seen which of these are most effective in engaging visitors in the informal learning experience that museums often seek. Whilst 40% of museums have noticed an increase in online visits (NEMO, 2020) and with the Royal Armouries online exhibition citing that 65% of visitors to their website were new (Ward, 2020), this does not necessarily equate to a measurement of the impact of presentation methods used. Therefore, more evaluation of these practices is needed in future to better understand online museum visitor behaviour (Skov and Ingwerson 2014). Organisations like MuseumNext have questioned what online exhibitions are providing to visitors, with Maria Ciaccheri (2020) arguing for the importance to ‘calibrate [online content] to meet human needs.’ Ciaccheri commented on the need to clearly define objectives of content, provide a variety of different learning approaches, and adhere to universal design principles. Similarly, in a study of over 100 Italian state museums that provided online content during their closure, Agostino et al. (2020) identified three main dilemmas of online delivery. These were (a) user engagement, which questions both what users and museums want from online content; (b) how much control and autonomy a visitor should have during their online visit; and (c) whether online tours should be free. Therefore, if such digital services are to continue, these considerations need to be addressed to ensure that online exhibitions understand and work for their audiences to the same level that physical exhibitions do.

However, whilst it is important for these improvements to be made in a more long‐term delivery of online content, this does not mean to say that the digital exhibitions presented during the COVID crisis were of no value. Organisations like Arts Council England have praised cultural organisations in their role in society of ‘staying connected through our sharing of creativity and culture’ (Henley, 2020a) which has been described as ‘profound’ (McMahon, 2020) thus demonstrating the important value of the online content produced during the lockdown. This is particularly impressive when considering the level of resource museums were operating with during the pandemic. NEMO (2020) reported that income losses were up to 75–80%, and with the Cultural Recovery Fund of £1.57 bn from the UK Government announced, it is evident that museums were, and still are, facing serious financial difficulties. Staff resource was also impacted, with many museums using the furlough scheme, but also with a third of museums worldwide being forced to reduce their staff (ICOM, 2020). Therefore, when considering the online exhibitions, it is important to analyse them in the context of this limited financial and personnel resource. The fact that such sophisticated presentation of online content was developed in a short space of time is inspiring, particularly since in many cases it successfully expressed the key themes of connection, embodiment and access.

With the growing realisation that ‘none of us will be returning to the pre‐pandemic world’ and so ‘our sector will need to change’ (Henley, 2020b), this is a ‘time to think about how a new vision for the museum sector might emerge’ (Fraser, 2020a). Therefore, the sector must consider several issues when developing more long‐term online content. However, with the problems of financial resource set to continue this will not be an easy task. Evidence of these changes can already been seen in relation to staff activity and priorities, with NEMO (2020) reporting that 30% of museums have changed staff tasks to provide services online. Despite this, there are concerns that staff teams are not fully equipped to handle such monumental changes, as evidenced by National Lottery Heritage Fund’s survey on Digital Skills for Heritage. In asking what skills and areas of digital technology organisations would like to explore, eight categories of activity emerged, including marketing and communications; creating content; and developing a digital strategy (Fraser, 2020b). This highlights the practical challenge of enabling the rise of digital content for museums, which will be difficult for the sector in such a stretched resource environment.

Alongside these practicalities, as this research evidences, there are also some conceptual challenges that organisations need to face. Whilst the content produced had great value in its time and place, this is now an opportunity, with a great sense of change emerging in the sector, to step back, reflect on, and take stock of online practices. If more attention is to be paid to online content and exhibitions, then these fundamental questions need to be considered to ensure institutions can continue to serve their audiences. These questions include: what is the value of the online exhibition? What should it do and not do? How is it different to a physical exhibition? How do audiences behave in online spaces? What do they expect? Future research into this area, evaluating how online exhibitions serve audiences, is thus needed. Only by refocusing attention and properly addressing these questions for both themselves and their audiences can museums continue to serve their vital role in their communities and society, both in physical spaces and in the digital sphere.

## Conclusion

COVID‐19 closures disrupted UK museum temporary exhibition programmes extensively, and institutions responded in a variety of ways, as evidenced by the 88 exhibitions that were due to open between March and June 2020. Despite the majority of museums either closing, extending, or postponing exhibitions, 21 exhibitions were identified as providing some form of online content. Through a detailed study of this content, it was evidenced how the pandemic was discussed in a slightly optimistic tone, with museums recognising their role in communities of providing hope and escapism in a time of uncertainty. The online exhibitions were presented in a variety of different ways using text, images, video, audio, and social media, and ranged from linear presentation which gave visitors little capacity to navigate freely, to the full freedom of a Virtual Reality tour. Themes of embodiment were identified, with frequent references made to the physical museum space, and there was a heightened sense of human connection through the visibility of curators, artists and researchers. Community engagement was also encouraged through social media use. These themes posed further questions of the value, role and identity of online exhibitions that was important to consider as museums continue such activities in the long term. Embedding this research within the wider literature concerning digital heritage, it is evident that the COVID crisis has been a pivotal moment for museums and the heritage sector more generally, having accelerated need and heightened the attention for relevant and meaningful digital practice. With this rising atmosphere of change on the horizon, it is important that museums consider such conceptual issues and evaluate audience needs rigorously when developing online offerings to maintain such cultural importance.

## Note

^1^

https://en.wikipedia.org/wiki/List_of_most_visited_museums_in_the_United_Kingdom

## Appendix 1 Data Sources

The below numbers are cited in text within “[ ]”.

1. *Young Rembrandt*, Ashmolean Museum, https://www.ashmolean.org/youngrembrandtonline
2. *Cranach: Artist and Innovator:* Compton Verney Art Gallery and Park, https://www.comptonverney.org.uk/cranach‐artist‐innovator/
3. *Langlands and Bell: Degrees of Truth*, Sir John Soanes Museum, https://www.soane.org/whats‐on/exhibitions/langlands‐bell‐degrees‐truth
4. *Edmund de Waal: Library of Exile*, British Museum, https://libraryofexile.infoteca.it/start
5. *The Enchanted Interior*, Guildhall Art Gallery
6. *50 Years of the Macrobert Award*, Science and Media Museum, https://www.scienceandmediamuseum.org.uk/whats‐on/50‐years‐macrobert‐award‐engineering‐innovation
7. *John Hitchens: Aspects of Landscape*, Southampton Art Gallery, https://www.southamptoncityartgallery.com/whats‐on/john‐hitchens/
8. *Florence Nightingale: Health in the Home,* Pickford’s House, https://www.derbymuseums.org/whats‐on/florence‐nightingale‐health‐in‐the‐home
9. *Skyscape*, Worcester Art Gallery, https://www.museumsworcestershire.org.uk/avada_portfolio/skyscape/
10. *Blitzed*, Nuneaton Museum
11. *Dawn of the Wonder Chicken,* Sedgwick Museum of Earth Sciences, https://wserv4.esc.cam.ac.uk/online‐exhibitions/index.php/Wonderchicken/dawn‐of‐the‐wonderchicken‐2/
12. *All Fired Up: A History of Fire Fighting in Essex*, Saffron Walden Museum, http://www.saffronwaldenmuseum.org/ follow What’s On > Current Exhibition
13. *Florence Nightingale Bicentenary: Inspiration to Genius,* Lotherton Museum, https://museumsandgalleries.leeds.gov.uk/virtual‐visit/florence‐nightingale‐online‐exhibition/
14. *Making Japan: Art, Culture, Life*, Lotherton Museum, https://museumsandgalleries.leeds.gov.uk/virtual‐visit/making‐japan‐online‐exhibition/
15. *Below the Salt*, Temple Newsam, https://museumsandgalleries.leeds.gov.uk/virtual‐visit/below‐the‐salt/?occurrence=2020‐04‐04
16. *Becoming Vanderbilt*, The Breakers, https://my.matterport.com/show/?m=Tge8ZGmYYro
17. *Into the Blue*, Tenby Museum, http://www.tenbymuseum.org.uk/a‐shop/
18. *Aberdeen Artists Society: Coming Home,* Aberdeen Art Museums
19. *A Curator’s Choice*, The Hunterian, https://www.gla.ac.uk/hunterian/visit/exhibitions/virtualexhibitions/acuratorschoice
20. *The Maguire Story*, Enniskillen Castle Museum
21. *Lisburn and the Second World War,* Irish Linen Centre, https://www.lisburnmuseum.com/virtual‐museum/ve75‐lisburn‐and‐the‐second‐world‐war/

**Table 1:** Exhibition sample by opening month

	Frequency	Percent
March	43	48.9
April	16	18.2
May	17	19.3
June	12	13.6
Total	88	100.0

**Table 2:** Exhibition sample by UK region

	Frequency	Percent
London	19	21.6
South West	8	9.1
South East	5	5.7
East Midlands	5	5.7
West Midlands	6	6.8
East of England	7	8.0
Yorkshire and the Humber	9	10.2
North East	5	5.7
North West	5	5.7
Wales	6	6.8
Scotland	9	10.2
Northern Ireland	4	4.5
Total	88	100.0

**Table 3:** Exhibition response to COVID‐19

	Frequency	Percent
Museum Closed	47	53.4
Extended	4	4.5
Postponed	16	18.2
Online Content	21	23.9
Total	88	100.0

## References

[cura12413-bib-0001] Agostino, D. , Arnaboldi, M. , & Lema, M. D. (2020). New development: COVID‐19 as an accelerator of digital transformation in public service delivery. Public Money and Management, 41(1), 1–4.

[cura12413-bib-0002] Bandelli, A. (2010). Virtual Spaces and Museums. In R. Parry (Ed.), Museums in a digital age (pp. 148–152). Routledge.

[cura12413-bib-0003] Battro, A. M. (2010). From Malraux’s imaginary museum to the virtual museum. In R. Parry (Ed.), Museums in a digital age (pp. 136–147). Routledge.

[cura12413-bib-0004] Ciaccheri, M. C. (2020). Do virtual tours in museums meet the real needs of the public? Observations and tips from a visitor studies perspective. MuseumNext. https://www.museumnext.com/article/do‐virtual‐tours‐in‐museums‐meet‐the‐real‐needs‐of‐the‐public‐observations‐and‐tips‐from‐a‐visitor‐studies‐perspective/

[cura12413-bib-0005] Dourish, P . (2001). Where the Action is: The Foundations of Embodied Interaction. Cambridge, MA: MIT Press.

[cura12413-bib-0006] Euston, N. (2020). Developing online exhibitions at Victoria Gallery & Museum. Museums Association. https://www.museumsassociation.org/museums‐journal/in‐practice/2020/09/developing‐online‐exhibitions‐at‐victoria‐gallery‐museum/?utm_campaign=1803911_04092020&utm_medium=email&utm_source=Museums%20Association&dm_i=2VBX,12NWN,7HEQ2T,444SI,1

[cura12413-bib-0007] Fraser, J. (2020a). Museum priorities. Curator: the Museum Journal, 63(2), 165–166.

[cura12413-bib-0008] Fraser, J. (2020b). Digital Attitudes and Skills for Heritage: What we have learned so far. Heritage Fund. https://www.heritagefund.org.uk/stories/digital‐attitudes‐and‐skills‐heritage‐what‐we‐have‐learned‐so‐far

[cura12413-bib-0009] Galani, A. , & Chalmers, M. (2010). Empowering the Remote Visitor: supporting social museum experiences among local and remote visitors. In R. Parry (Ed.), Museums in a Digital Age (pp. 159–169). Routledge.

[cura12413-bib-0010] Hall, E. T. (1969). The Hidden Dimension. New York: Doubleday and Company.

[cura12413-bib-0011] Henley, D. (2020a). Kindness in a time of crisis. Arts Council England. https://www.artscouncil.org.uk/blog/kindness‐time‐crisis

[cura12413-bib-0012] Henley, D. (2020b). What comes next? Arts Council England. https://www.artscouncil.org.uk/blog/what‐comes‐next

[cura12413-bib-0013] Huhtamo, E. (2010). On the origins of the virtual museum. In R. Parry (Ed.), Museums in a digital age (pp. 121–135). Routledge.

[cura12413-bib-0014] International Council of Museums (ICOM) (2020). Museums, museum professionals and COVID‐19. https://icom.museum/wp‐content/uploads/2020/05/Report‐Museums‐and‐COVID‐19.pdf

[cura12413-bib-0015] Johnson, M . (1987). The Body in the Mind: The Bodily Basis of Meaning, Imagination, and Reason. Chicago: University of Chicago Press.

[cura12413-bib-0016] Johnson, M. (2007). The Meaning of the Body: Aesthetics of Human Understanding. Chicago: University of Chicago Press.

[cura12413-bib-0017] Kenderdine, S. (2015). Embodiment, entanglement and immersion in digital cultural heritage. In S. Schreibman , R. Siemens , & J. Unsworth (Eds.) A new companion to digital humanities (pp. 22–41). John Wiley and Sons.

[cura12413-bib-0018] Lakoff, G. , & Johnson, M. (1980). Metaphors we Live by. Chicago: University of Chicago Press.

[cura12413-bib-0019] Lakoff, G. , & Johnson, M. (1999). Philosophy in the Flesh: The Embodied Mind and its Challenge to Western Thought. New York: Basic Books.

[cura12413-bib-0020] Lin, A. C. H. , & Gregor, S. (2006). Designing websites for learning and enjoyment: A study of museum experiences. Research in Open and Distance Learning, 7(3), 1–21.

[cura12413-bib-0021] Marty, P. F. (2008). Museum Websites and Museum Visitors: digital museum resources and their use. Museum Management and Curatorship, 23(1), 81–99.

[cura12413-bib-0022] Maxwell, P. (2020). The rise of the virtual gallery tour: What works and what doesn’t (yet). Frame. https://www.frameweb.com/news/virtual‐gallery‐tour‐covid‐19‐crisis

[cura12413-bib-0023] McMahon, J. (2020). Getting creative for health and wellbeing. Arts Council England. https://www.artscouncil.org.uk/blog/getting‐creative‐health‐and‐wellbeing

[cura12413-bib-0024] Meehan, N. (2020). Digital museum objects and memory: Postdigital materiality, aura, and value. Curator: The Museum Journal, 1–17.

[cura12413-bib-0025] Network of European Museum Organisations (NEMO) (2020). Survey on the impact of the COVID‐19 situation on museums in Europe. https://www.ne‐mo.org/fileadmin/Dateien/public/NEMO_documents/NEMO_Corona_Survey_Results_6_4_20.pdf

[cura12413-bib-0026] Parry, R. (2005). Digital Heritage and the Rise of Theory in Museum Computing. Museum Management and Curatorship, 20(4), 333–348.

[cura12413-bib-0027] Parry, R. (2007). Recoding the museum: Digital heritage and the technologies of change. Routledge.

[cura12413-bib-0028] Parry, R. (2010). The practice of digital heritage and heritage of digital practice. In R. Parry (Ed.), Museums in a digital age (pp. 2–7). Routledge.

[cura12413-bib-0029] Slater, M. (1999). Measuring Presence: A Response to the Witmer and Singer Presence Questionnaire. Presence: Teleoperators and Virtual Environments, 8(5), 560–565.

[cura12413-bib-0030] Rahaman, H. (2018). Digital heritage interpretation: A conceptual framework. Digital Creativity, 29(2–3), 208–234.

[cura12413-bib-0031] Taylor‐Jones, N. (2020). Creating virtual tours at the Museum of Gloucester. Museums Association. https://www.museumsassociation.org/museums‐journal/in‐practice/2020/09/creating‐virtual‐tours‐at‐the‐museum‐of‐gloucester/?utm_campaign=1804920_07092020&utm_medium=email&utm_source=Museums%20Association&dm_i=2VBX,12OOO,7HEQ2T,4487U,1

[cura12413-bib-0032] Tuan, Y‐F . (2001). Space and Place: The Perspective of Experience. Minneapolis: University of Minnesota Press.

[cura12413-bib-0033] United Nations Educational, Scientific and Cultural Organisations (UNESCO) (2020). Museums around the world in the face of COVID‐19. https://unesdoc.unesco.org/ark:/48223/pf0000373530

[cura12413-bib-0034] Walsh, D. , Hall, M. M. , Clough, P. , & Foster, J. (2020). Characterising online museum users: a study of the national museums liverpool museum website. International Journal on Digital Libraries, 21, 75–87.

[cura12413-bib-0035] Ward, A. (2020). Turning physical exhibitions into digital ones. Museums Association. https://www.museumsassociation.org/museums‐journal/in‐practice/2020/09/turning‐physical‐exhibitions‐into‐digital‐ones/?utm_campaign=1802352_02092020%20MA%20newsletter&utm_medium=email&utm_source=Museums%20Association&dm_i=2VBX,12MPC,7HEQ2T,43ZGM,1

[cura12413-bib-0036] Wellington, S. , & Oliver, G. (2015). Reviewing the digital heritage landscape: The intersection of digital media and museum practice. In C. McCarthy (Ed.), The international handbooks of museum studies: Museum practice (pp. 577–598). John Wiley & Sons.

[cura12413-bib-0037] Williams, P. (1987). A brief history of museum computerization. Museum Studies Journal, 3(1), 58–65.

